# A carnosine analog with therapeutic potentials in the treatment of disorders related to oxidative stress

**DOI:** 10.1371/journal.pone.0215170

**Published:** 2019-04-09

**Authors:** Rita Rezzani, Gaia Favero, Matteo Ferroni, Claudio Lonati, Mohammed H. Moghadasian

**Affiliations:** 1 Anatomy and Physiopathology Division, Department of Clinical and Experimental Sciences, University of Brescia, Viale Europa, Brescia, Italy; 2 Interdipartimental University Center of Research “Adaption and Regeneration of Tissues and Organs- (ARTO)”, University of Brescia, Brescia, Italy; 3 Department of Information Engineering, University of Brescia, Via Branze, Brescia, Italy; 4 CNR-IMM Bologna Section - Via Gobetti, Bologna, Italy; 5 Department of Human Nutritional Sciences, University of Manitoba and Canadian Centre for Agri-Food Research in Health and Medicine, St. Boniface Hospital Research Centre, Winnipeg, MB, Canada; National Institutes of Health, UNITED STATES

## Abstract

Interactive relationships among metabolism, mitochondrial dysfunction and inflammation at skeletal muscle level play a key role in the pathogenesis of disorders related to oxidative stress. Mitochondrial dysfunction and oxidative stress result in cellular energy deficiency, inflammation and cell death inducing a vicious cycle that promotes muscle wasting. The histidine-containing dipeptides, carnosine and anserine, are carbonyl scavengers whose cytoprotective contributions extend beyond the antioxidant defence, but the physiological meaning of these capacities is actually limited. In the present study, we compared and investigated the potential protective effects of three different histidine-containing dipeptides: carnosine, anserine and carnosinol, a carnosine-mimetic new compound, against oxidative stress induction in rat L6 skeletal muscle cells. The hydrogen peroxide induced-oxidative stress significantly altered cell morphology, induced apoptosis, oxidative stress and inflammation, decreased mitochondrial peroxisome proliferator-activated receptor gamma coactivator-1α (PGC-1α)/sirtuin3 pathway and the antioxidant system. Notably, all three investigated dipeptides in the present study, with a different extent and in a concentration-dependent manner, reduced myotube oxidative stress, apoptosis and inflammation. The present study underlined that carnosinol, maintaining the safety condition of carnosine and anserine, was the more efficient studied dipeptide in the preservation of mitochondrial environment mediated by PGC-1α and sirtuin3 expression and thereby in the reduction of oxidative stress-related alterations in this *in vitro* skeletal muscle model. Furthermore, we observed that carnosinol’s antioxidant effects are not blocked inhibiting sirtuin3, but are maintained with almost the same extend, indicating its multiple capacities of reactive carbonyl species-scavenging and of mitochondrial modulation through PGC-1α. In conclusion, carnosinol retained and surpassed the efficacy of the well-known investigated histidine-containing dipeptides improving oxidative stress, inflammation and also cell metabolism and so becoming a greatly promising therapeutic carnosine derivate.

## Introduction

Carnosine and anserine are versatile histidine-containing dipeptides (HCDs) identified in vertebrates, including horses, greyhounds, camels and humans [1]. HCDs are stored in several tissues with the highest concentration occurring in skeletal muscle [2]. These dipeptides have several important physiological properties and, in particular, carnosine plays many roles in maintaining health, including antioxidant activity [3]. Anserine has similar effects, acting as an antioxidant and carbonyl scavenger [4] and affecting renal sympathetic nerve activity and blood pressure because, in kidney, it is expressed in concentration two times higher than those of carnosine [5].

It has been suggested that HCDs could act as anti-ageing agents increasing the number of times that the cells can divide and apparently rejuvenating senescent cells [6]. Although these results are very exciting, there are not enough studies *in vitro* and in cultured cell models and little is known about the physiological meaning of these capacities. Moreover, it is not clear how these HCDs can affect lifespan and ageing in different tissues.

Recently, a reduced derivative of carnosine, carnosinol, has been identified displaying selectivity for reaction with reactive carbonyl species (RCS), i.e. sugar-and lipid derived aldehydes [7]. These Authors studied the pathological role of RCS in obesity with the pharmacological evaluation of carnosinol. They suggested that carnosinol is the most promising carnosine derivate that has been synthesized until now, even if the studies are at the beginning.

However, whereas *in vivo* and *in vitro* antioxidant activities of carnosine and anserine have been evaluated [8–10], the potential effects of carnosinol are not yet still investigated.

Thus, the aim of this study is to present the morphofunctional evaluation of carnosinol effects in L6 skeletal muscle cells *in vitro* in order to validate the use of this novel RCS-scavenging carnosine derivate in physiological and not physiological conditions. We evaluated cell viability, the potential effect of carnosinol against apoptosis, inflammation and oxidative stress-induced by hydrogen peroxide (H_2_O_2_) incubation, as previously reported by our group [11]. In particular, we demonstrated that carnosinol maintains the safety condition of carnosine/anserine and its more effective than the other studied HCDs.

## Materials and methods

### Cell culture

L6 rat skeletal myoblasts, obtained from the Experimental Zooprophylactic Institute of Lombardy and Emilia Romagna “Bruno Ubertini” (BS CL 134—Source: American Type Culture Collection, Rockville, Md, USA), were cultured in Dulbecco’s modified Eagle’s medium (DMEM) supplemented with 10% heat-inactivated fetal bovine serum (FBS), penicillin (100 U/ml) and streptomycin (100 μg/ml) and incubated at 37°C in a humidified atmosphere with 5% carbon dioxide and 95% air atmosphere. Details about cell culture methods and myogenic differentiation have been previously reported by [11].

The L6 myoblasts were plated in culture 6-wells, induced to differentiate and randomly divided into the following experimental groups: control without any treatment, H_2_O_2_ treatment, carnosinol incubation, carnosine incubation, anserine incubation, carnosinol pre-incubation followed by H_2_O_2_ treatment, carnosine pre-incubation followed by H_2_O_2_ treatment, and anserine pre-incubation followed by H_2_O_2_ treatment. In detail, the pre-incubations of 24 hours with powder of pure carnosinol (C_9_H_16_N_4_O_4_), carnosine (C_9_H_14_N4O_3_) or anserine (C_10_H_15_N_4_O_3_) (the molecules were kindly provided by Flamma S.p.A., Chignolo d’Isola, Bergamo, Italy) were dissolved in differentiation medium at three increasing concentrations for each molecule (10 mM, 20 mM or 30 mM). In the experimental group of induced-oxidative stress, the myotubes were treated with H_2_O_2_ at the final concentration of 50 μM for 1 hour, as previously described by [11, 12]. At the aim to evaluate in deep the mechanism of action of carnosinol, in the second series of experiments, L6 rat skeletal myoblasts were exposed to 2 μM of sirtuin3 (SIRT3) inhibitor (AGK7; Cayman Chemical, Ann Arbor, MI, USA) either vehicle (DMSO) for 16 hours [13], then were incubated with carnosinol at the higher concentration studied (30 mM) and followed by H_2_O_2_-treated.

### Cell viability assay

At the end of treatments, the myotubes were collected and resuspended in phosphate buffer solution (PBS) containing 0.4% trypan blue. Counts of viable (unstained) and non-viable (blue-stained) cells were made using a light microscope with a haemocytometer, and the percentage of viable cells was calculated as previously reported by [11, 14]. The assessment of cell viability was carried out by two independent observers blinded to cell treatments. In the case of dispute concerning interpretation, the case was reconsidered until reaching an agreement.

### Tunel assay

Internucleosomal DNA fragmentation is a hallmark of apoptosis in mammalian cells. The TUNEL reaction (terminal-mediated dUTP nick end labelling) was used to analyse DNA fragmentation in all experimental cell groups investigated in the present study. The TUNEL detection kit (Gene Tex Inc., Irvine, CA, USA) utilizes terminal deoxynucleotidyl transferase (TdT) to catalyze incorporation of fluorescein-12-dUTP at the free 3’-hydroxyl ends of the fragmented DNA and then the fluorescein-labeled DNA was observed by fluorescence microscopy. The procedure was carried out according to the manufacturer’s instructions. Apoptotic cells were examined at 400× magnification over 20 fields per experimental group and expressed as percentage of tunnel positive cells. The assessment of cell apoptosis was carried out by two independent observers blinded to cell treatments. In the case of dispute concerning interpretation, the case was reconsidered until reaching an agreement.

### Scanning electron microscopy

Control and treated myotubes were cultivated on glass coverslips in 6-wells and then the monolayers were dehydratated with ethanol and left to dry overnight at room temperature. No coating with gold was performed. The samples were observed in high-vacuum condition (about 10–7 mbar pressure range) by scanning electron microscopy (SEM, Field-Emission LEO 1525) equipped with conventional Everhart-Thorley and In-Lens detectors for secondary-electron imaging and operated in the 3–10 keV range of beam energy. Operation of the SEM at low beam energy, fast scanning modality and tilting of the stage, prevented the specimens from electrostatic charging and allowed an effective observation of the myotube morphology at different magnification.

### Immunofluorescence and immunomorphometrical assay

At the end of the treatment period, L6 cells of each experimental group were fixed in 4% buffered paraformaldehyde for 10 minutes, washed in PBS and incubated in 0.3% bovine serum albumin for 1 hour at room temperature and then overnight at 4°C with the following antibodies: goat polyclonal catalase antibody (CAT; diluted 1:200; sc34285—Santa Cruz Biotechnology Inc., Dallas, TX, USA); rabbit cyclooxygenase2 antibody (COX2; diluted 1:300; ab15191—Abcam, Cambridge, UK); rabbit polyclonal peroxisome proliferator-activated receptor gamma coactivator-1α (PGC-1α) (dilution 1:300; ab191838—Abcam, Cambridge, UK); rabbit polyclonal SIRT3 (diluted 1:150; ab189860—Abcam, Cambridge, UK) and rabbit polyclonal superoxide dismutase2 (SOD2) (dilution 1:300; ab13534—Abcam, Cambridge, UK). Thereafter, the myotubes were labelled with the respective conjugated secondary antibody anti-goat or anti-rabbit Alexa Fluor 488 or anti-rabbit Alexa Fluor 546 (diluted 1:200; Life Technologies, Grand Island, NY, USA). Finally, the cells were counterstained with 4',6-diamidino-2-phenylindole (DAPI) [14–16]. The cell immunofluorescence assays were observed with a fluorescent microscope (i50 Eclipse, Nikon, Düsseldorf, Germany) as previously described by [17, 18]. The control for each immunofluorescence was performed by omitting the primary antibody and in the presence of isotype-matched total immunoglobulin G.

Immunopositivity (staining intensity) of each immunofluorescence analyses was computed by two independent observers blinded to the cell treatments using an image analyser (Image Pro Premier 9.1, Media Cybernetics, Rockville, MD, USA) as previously described [11, 14].

### Western blot evaluation

The cell homogenates were loaded into 10% SDS-polyacrylamide gels and subjected to electrophoresis. The separated proteins were transferred to nitrocellulose membranes and then incubated with bovine albumine serum solution for 1 hour, followed by overnight incubation at 4°C with the following antibodies: mouse monoclonal β-actin antibody (diluted 1:5000; AC5441 –Sigma Aldrich, St. Louis, MO, USA), goat polyclonal CAT antibody (CAT; diluted 1:1000; sc34285—Santa Cruz Biotechnology Inc., Dallas, TX, USA), rabbit polyclonal PGC-1α (dilution 1:2500; ab191838—Abcam, Cambridge, UK); rabbit polyclonal SOD2 (diluted 1:2000; ab13534—Abcam, Cambridge, UK), or with rabbit polyclonal SIRT3 (diluted 1:1500; ab189860—Abcam, Cambridge, UK). Protein detection was carried out using secondary infrared fluorescent dye conjugated antibodies absorbing at 800 nm or 700 nm. The blots were visualized using an Odyssey Infrared Imaging Scanner (Li-Cor Inc., Lincoln, USA).

### Statistical analysis

The data were pooled to calculate a mean value and results were expressed as the mean±standard deviation. Data for multiple variable comparisons were analyzed by ANOVA corrected Bonferroni test, with significance set at p≤0.05.

## Results

**Fig 1:**
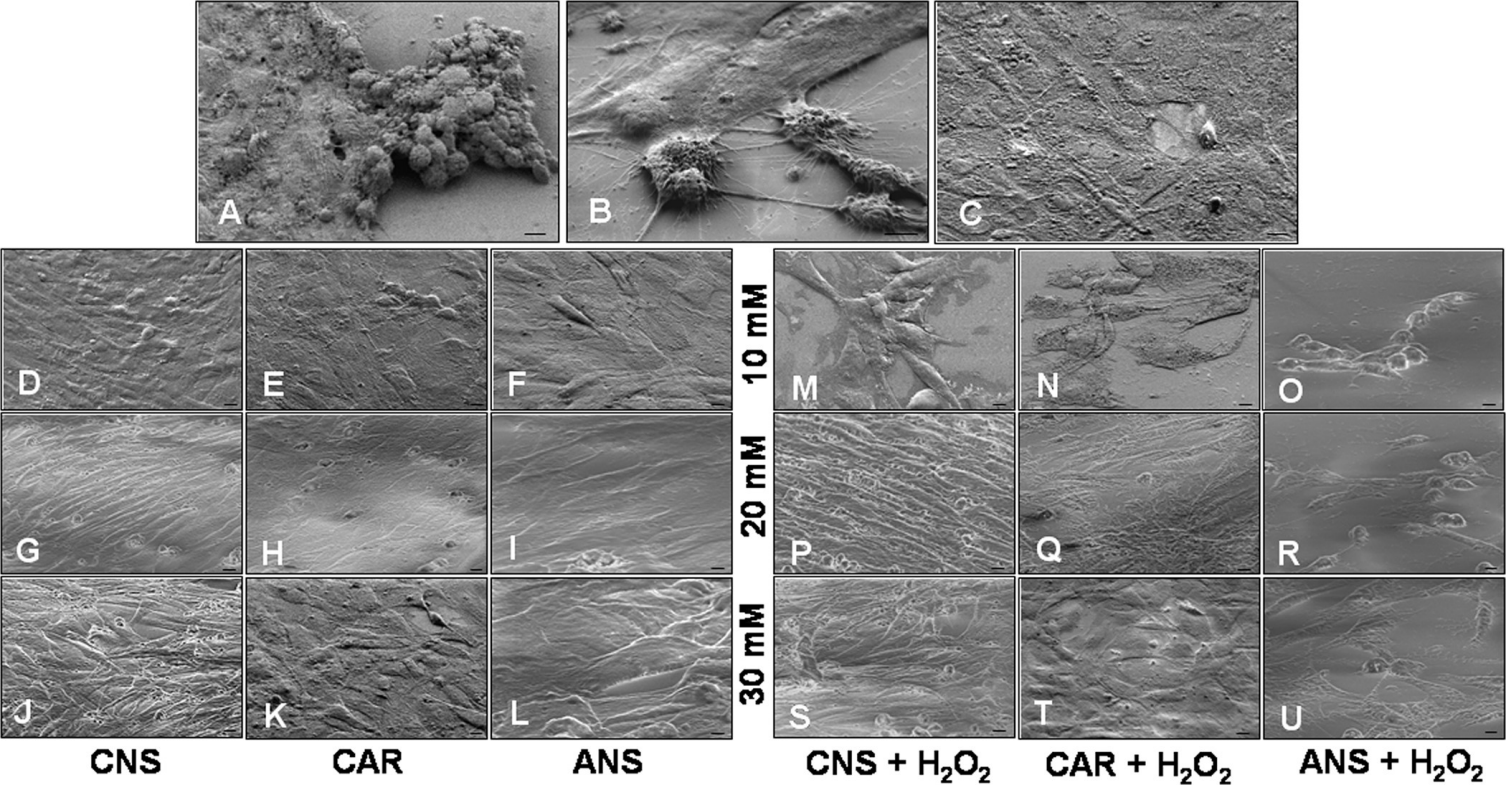
Myotube morphology evaluation. Scanning electron microscopy photomicrographs showing myotubes treated with hydrogen peroxide—H_2_O_2_ (A, B), untreated control myotubes—CTR (C), myotubes pre-incubated with carnosinol—CNS (D, G, J), carnosine—CAR (E, H, K) or anserine—ANS (F, I, L) at the concentrations of 10 mM (D-F), 20 mM (G-I) and 30 mM (J-L), myotubes pre-incubated with carnosinol followed by hydrogen peroxide treatment—CNS + H_2_O_2_ (M, P, S), pre-incubated with carnosine followed by hydrogen peroxide treatment—CAR + H_2_O_2_ (N, Q, T) or pre-incubated with anserine followed by hydrogen peroxide treatment—ANS + H_2_O_2_ (O, R, U) at the concentration of 10 mM (M-O), 20 mM (P-R) and 30 mM (S-U). Bar equal: 10 μm.

The oxidative stress induced by incubation of L6 myotubes with H_2_O_2_, as previously observed also by [11], exhibited a typical morphological pattern of apoptosis: cells with small size and several membrane blebbing, while few cells were apparently preserved. In particular, numerous H_2_O_2_-treated myotubes looked rounded and detached (**Fig 1A and 1B**) respect to untreated control cells that appeared elongated and with a rod shape, except for few dead cells that showed a reduced size (**Fig 1C**). Cells incubated only with carnosinol, carnosine or anserine, independently of the concentration, showed the same normal morphological pattern (**Fig 1D–1L**) observed in untreated control myotubes. Interestingly, H_2_O_2_-reduction of cell viability and cell size were prevented, but to a different extent, by carnosinol, carnosine or anserine pre-incubation (**Fig 1M–1U**). The pre-incubation of H_2_O_2_-treated cells with 10 mM carnosinol (**Fig 1M**) or 10 mM carnosine (**Fig 1N**) showed prevalently scattered and round cells with membrane blebs; nevertheless, 10 mM carnosinol pre-incubation induced an increasing cell survival rate, not evident after pre-incubation of H_2_O_2_-incubated cells with carnosine 10 mM or anserine 10 mM and 20 mM (**Fig 1O and 1R**). These latter showed almost the same pattern of H_2_O_2_-incubated cells and just a weak preservation of myotubes morphology with a reduction of membrane blebs at the concentration of 30 mM (**Fig 1U**). Remarkably, the pre-incubation of H_2_O_2_-treated cells with carnosinol at the concentrations of 20 mM and 30 mM preserved, greatly and in a concentration-dependent manner, cell shape and size (**Fig 1P and 1S**) showing a cell morphology comparable to untreated control and preserving cell shape and size also respect to concentration-matched H_2_O_2_-treated cells pre-incubated with carnosine, which presented some round dead cells with blebs (**Fig 1Q and 1T**).

**Fig 2:**
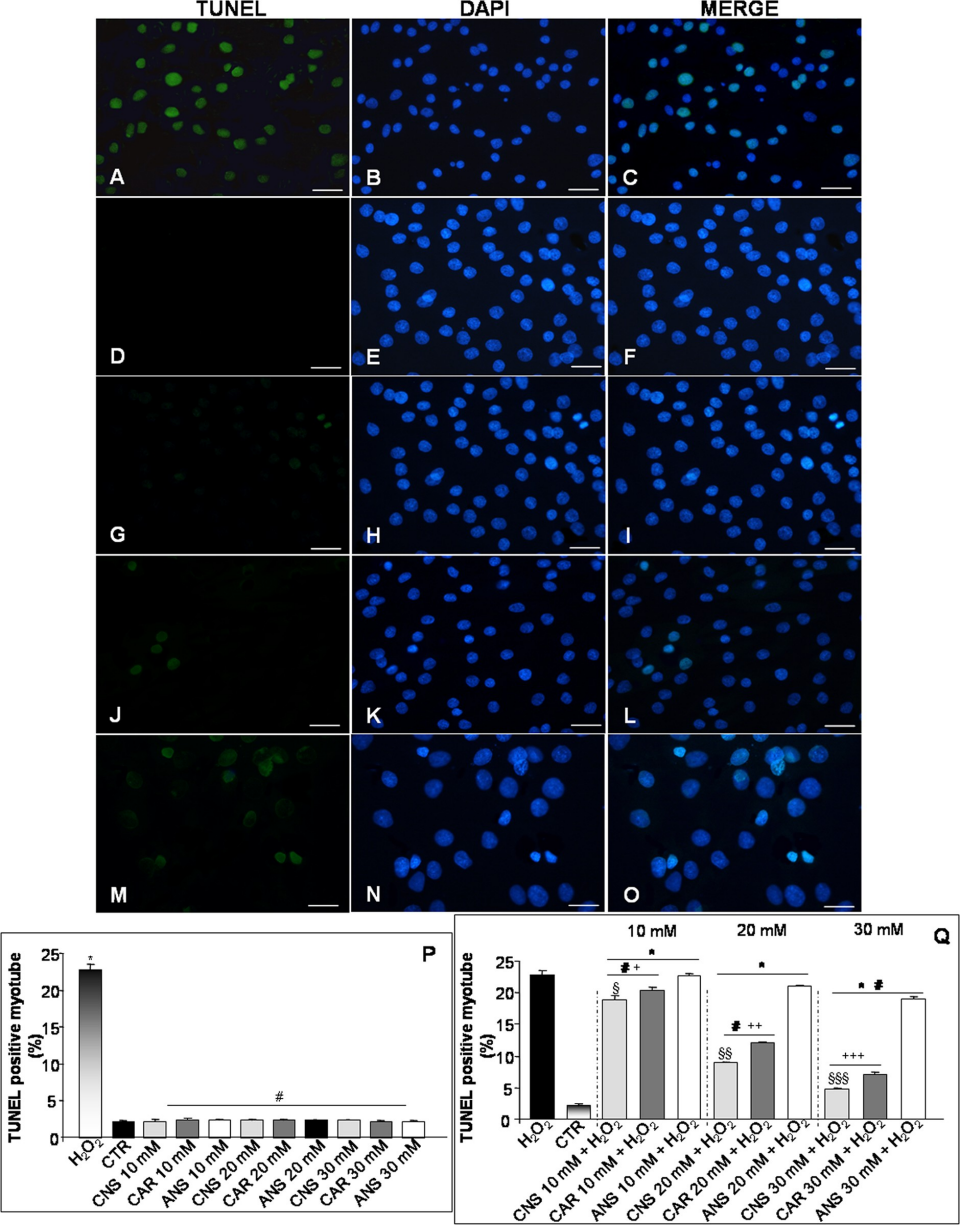
Myotube apoptotic evaluation. Photomicrographs of TUNEL assay (green staining) showing myotubes treated with hydrogen peroxide—H_2_O_2_ (A-C), untreated control myotubes—CTR (D-F), myotubes pre-incubated with carnosinol (30 mM) followed by hydrogen peroxide treatment—CNS + H_2_O_2_ (G-I), pre-incubated with carnosine (30 mM) followed by hydrogen peroxide treatment—CAR + H_2_O_2_ (J-L) and pre-incubated with anserine (30 mM) followed by hydrogen peroxide treatment—ANS + H_2_O_2_ (M-O). DAPI (blue staining) is used to locate the nuclei of the cells. Bar equal: 20 μm. The graphs (P and Q) summarize the quantitative analysis of TUNEL positive myotubes of all the experimental cell groups. * p≤0.05 *vs* CTR; # p≤0.05 *vs* H_2_O_2;_ § p≤0.05 *vs* CAR + H_2_O_2_ 10 mM; §§ p≤0.05 *vs* CAR + H_2_O_2_ 20 mM; §§§ p≤0.05 *vs* CAR + H_2_O_2_ 30 mM; + p≤0.05 *vs* ANS + H_2_O_2_ 10 mM; ++ p≤0.05 *vs* ANS + H_2_O_2_ 20 mM and +++ p≤0.05 *vs* ANS + H_2_O_2_ 30 mM.

To confirm that the reduction of cell viability was due to apoptosis, we used the TUNEL assay. In particular, TUNEL evaluation showed that apoptotic cells increased significantly after H_2_O_2_ incubation (**Fig 2A–2C**) respect to untreated control myotubes that showed a very low/absent presence of apoptotic cells (**Fig 2D–2F**). Remarkably, myotubes incubated only with each investigated HCDs showed absence/very weak presence of apoptotic cells, comparable to untreated control myotubes. The pre-incubation of H_2_O_2_-treated cells with carnosinol or carnosine at the concentration of 10 mM or with anserine at all the studied concentrations showed a moderate presence of apoptotic cells. Interestingly, the pre-incubation of H_2_O_2_-treated cells with carnosinol 20 mM or 30 mM showed a significant reduction of apoptotic cells as compared with H_2_O_2_-incubated cells. This reduction is observable also in 20 mM and 30 mM carnosine pre-incubated H_2_O_2_-treated cells, but with a lower extent. In **Fig 2G–2O** were reported representative TUNEL staining photomicrographs of H_2_O_2_-treated myotubes pre-incubated with the higher concentration studied of carnosinol (**Fig 2G–2I**), carnosine (**Fig 2J–2L**) and anserine (**Fig 2M–2O**). All the above reported observations are confirmed also by TUNEL apoptotic positive cells evaluation and are summarized in **Fig 2P and 2Q.**

**Fig 3:**
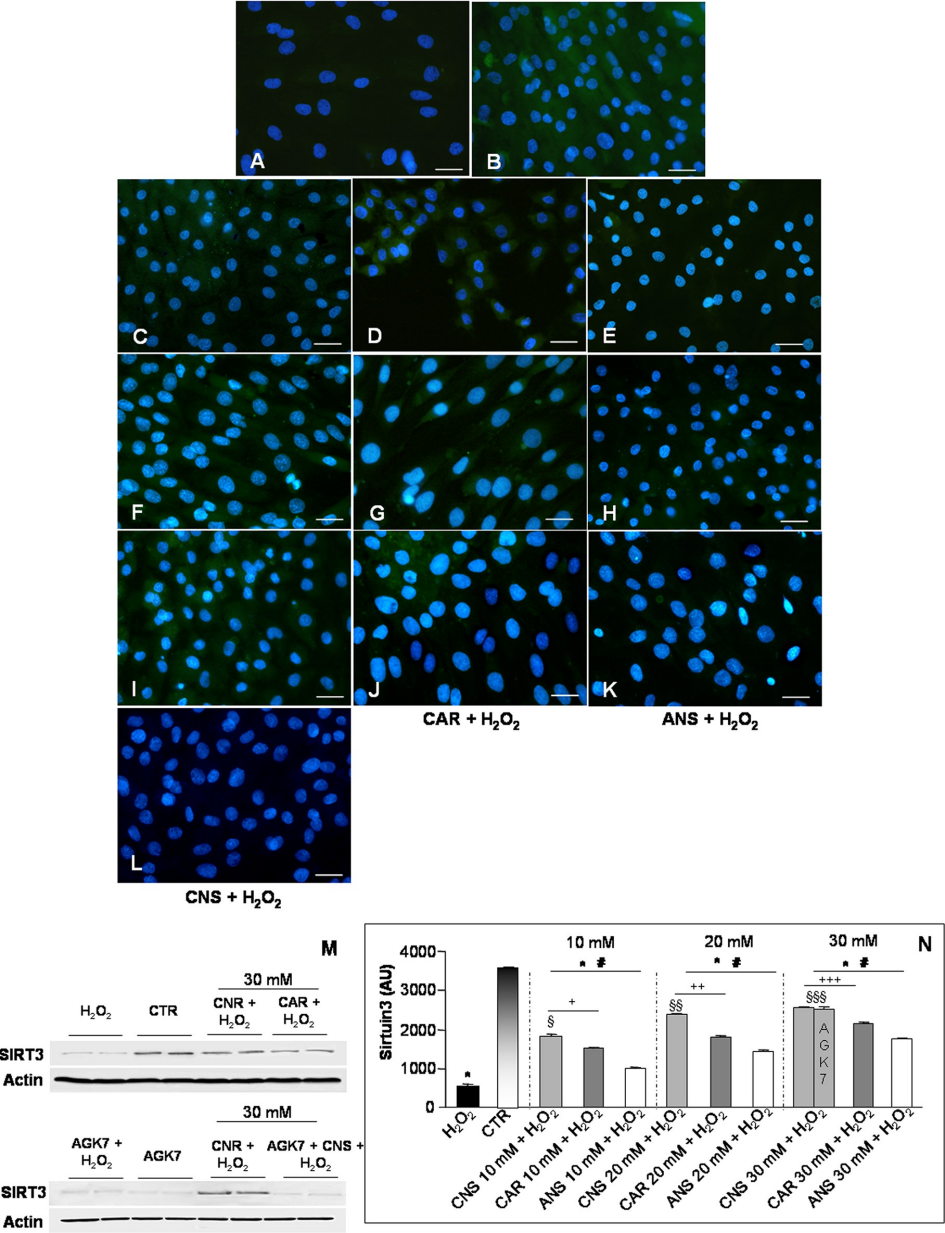
Sirtuin3 expression. Immunofluorescence photomicrographs of sirtuin3 (SIRT3—green staining) of myotubes treated with hydrogen peroxide—H_2_O_2_ (A), untreated control myotubes—CTR (B), myotubes pre-incubated with carnosinol followed by H_2_O_2_-treatment—CNS + H_2_O_2_ (C, F, I), pre-incubated with carnosine followed by H_2_O_2_-treatment—CAR + H_2_O_2_ (D, G, J), pre-incubated with anserine followed by H_2_O_2_-treatment—ANS + H_2_O_2_ (E, H, K) at the concentrations of 10 mM (C-E), 20 mM (F-H) and 30 mM (I-K) and myotubes incubated with AGK7 then with carnosinol (30 mM) and followed by H_2_O_2_-treatment—AGK7 + CNS + H_2_O_2_ (L). Bar equal: 20 μm. (M) Representative western blot showing SIRT3 level in myotubes treated with hydrogen peroxide—H_2_O_2_, untreated control myotubes—CTR, myotubes pre-incubated with carnosinol (30 mM) followed by H_2_O_2_-treatment—CNS + H_2_O_2_, myotubes pre-incubated with carnosine (30 mM) followed by H_2_O_2_-treatment—CAR + H_2_O_2_, myotubes incubated with AGK7 and then with hydrogen peroxide—AGK7 + H_2_O_2_, myotubes incubated with only AGK7—AGK7 and myotubes incubated with AGK7 then with carnosinol (30 mM) and followed by H_2_O_2_-treatment—AGK7 + CNS + H_2_O_2_. (N) The graph summarizes the quantitative analysis of SIRT3 immunopositivity. * p≤0.05 *vs* CTR; # p≤0.05 *vs* H_2_O_2;_ § p≤0.05 *vs* CAR + H_2_O_2_ 10 mM; §§ p≤0.05 *vs* CAR + H_2_O_2_ 20 mM; §§§ p≤0.05 *vs* CAR + H_2_O_2_ 30 mM; + p≤0.05 *vs* ANS + H_2_O_2_ 10 mM; ++ p≤0.05 *vs* ANS + H_2_O_2_ 20 mM and +++ p≤0.05 *vs* ANS + H_2_O_2_ 30 mM.

SIRT3 is an important regulator of oxidative stress and it is interestingly involved in protection against age-related diseases [19]. We observed that SIRT3 was absent or very weakly expressed in H_2_O_2_-treated cells (**Fig 3A**), while the expression of this deacetylase was moderate in the untreated control myotubes (**Fig 3B**). The incubation of L6 cells with only carnosinol, carnosine or anserine at the concentrations of 10 mM, 20 mM and 30 mM showed no difference in SIRT3 expression respect to control myotubes (**S1A Fig**). Remarkably, the pre-incubation of H_2_O_2_-treated myotubes with carnosinol showed a moderate/strong and concentration-dependent expression of SIRT3 (**Fig 3C, 3F and 3I**) respect to a weak/moderate expression in H_2_O_2_-treated cells pre-incubated with carnosine (**Fig 3D, 3G and 3J**) or to a weak expression in H_2_O_2_-treated cells pre-incubated with anserine (**Fig 3E, 3H and 3K**). In particular, increased SIRT3 expression was concentration-dependent in all the pre-treated groups and it was higher in H_2_O_2_-treated cells pre-incubated with carnosinol at 30 mM. As expected, AGK7 incubation induced a significant decrease in SIRT3 expression (almost absent) in H_2_O_2_-treated cells pre-incubated with carnosinol at the concentration of 30 mM (**Fig 3L**), but also in H_2_O_2_-treated myotubes or in control cells. The above reported observations are confirmed also by western blot analyses (**Fig 3M**). **Fig 3N** summarized SIRT3 immunopositivity evaluation.

**Fig 4:**
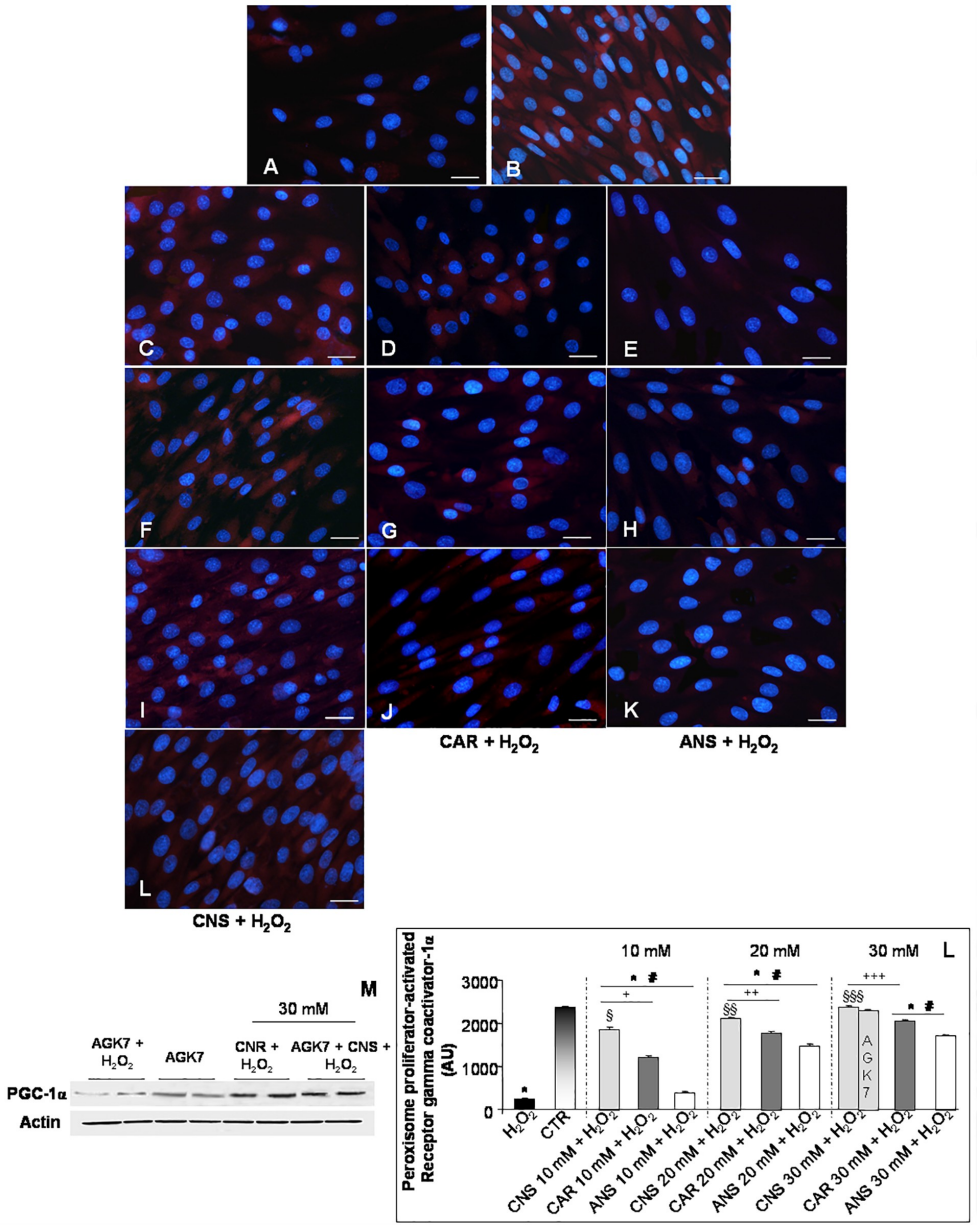
Peroxisome proliferator-activated receptor gamma coactivator-1α expression. Immunofluorescence photomicrographs of peroxisome proliferator-activated receptor gamma coactivator-1alpha (PGC-1α - red staining) of myotubes treated with hydrogen peroxide—H_2_O_2_ (A), untreated control myotubes—CTR (B), myotubes pre-incubated with carnosinol followed by H_2_O_2_-treatment—CNS + H_2_O_2_ (C, F, I), pre-incubated with carnosine followed by H_2_O_2_-treatment—CAR + H_2_O_2_ (D, G, J), pre-incubated with anserine followed by H_2_O_2_-treatment- ANS + H_2_O_2_ (E, H, K) at the concentrations of 10 mM (C-E), 20 mM (F-H) and 30 mM (I-K) and myotubes incubated with AGK7 then with carnosinol (30 mM) and followed by H_2_O_2_-treatment—AGK7 + CNS + H_2_O_2_ (L). Bar equal: 20 μm. (M) Representative western blot showing PGC-1α level in myotubes incubated with AGK7 and then with hydrogen peroxide—AGK7 + H_2_O_2_, myotubes incubated with only AGK7—AGK7, myotube pre-incubated with carnosinol (30 mM) followed by H_2_O_2_-treatment—CNS + H_2_O_2_ and myotubes incubated with AGK7 then with carnosinol (30 mM) and followed by H_2_O_2_-treatment—AGK7 + CNS + H_2_O_2_. (N) The graph summarizes the quantitative analysis of PGC-1α immunopositivity. * p≤0.05 *vs* CTR; # p≤0.05 *vs* H_2_O_2;_ § p≤0.05 *vs* CAR + H_2_O_2_ 10 mM; §§ p≤0.05 *vs* CAR + H_2_O_2_ 20 mM; §§§ p≤0.05 *vs* CAR + H_2_O_2_ 30 mM; + p≤0.05 *vs* ANS + H_2_O_2_ 10 mM; ++ p≤0.05 *vs* ANS + H_2_O_2_ 20 mM and +++ p≤0.05 *vs* ANS + H_2_O_2_ 30 mM.

Furthermore, we investigated also the expression of the mitochondrial PGC-1α, due to it controls cellular metabolic adaptation to environmental, mitochondrial biogenesis and oxidative stress and it is expressed also in health skeletal muscle [20]. In the present study, H_2_O_2_-treated myotubes showed a very weak PGC-1α expression (**Fig 4A**) respect to a strong expression in untreated control cells (**Fig 4B**). The incubation of L6 myotubes with only carnosinol, carnosine or anserine at the concentrations of 10 mM, 20 mM and 30 mM showed no difference in PGC-1α expression respect to control myotubes (**S1B Fig**). Notably, PGC-1α expression was, significantly and in concentration-dependent manner, increased after pre-incubation of H_2_O_2_-treated myotubes with carnosinol showing a moderate/strong expression (**Fig 4C, 4F and 4I**) respect to a weak/moderate and concentration-dependent expression in H_2_O_2_-treated cells pre-incubated with carnosine (**Fig 4D, 4G and 4J**) or to a weak expression in H_2_O_2_-treated cells pre-incubated with anserine (**Fig 4E, 4H and 4K**). Interestingly, myotubes incubated with AGK7 then with carnosinol (30 mM) and followed by H_2_O_2_-treatment maintained a moderate/strong PGC-1α expression (**Fig 4L**), as observed in myotubes pre-incubated with carnosinol (30 mM) and then H_2_O_2_-treated. Furthermore, the myotube incubated with AGK7 and then treated with H_2_O_2_ showed a very weak PGC-1α expression, as observed in H_2_O_2_-treated cells. Interestingly, myotubes incubated only with AGK7 showed a significant reduction of PGC-1α expression compared to the respective experimental group not treated with the SIRT3 inhibitor. The above reported observations are confirmed also by western blot analyses (**Fig 4M**). **Fig 4N** summarized PGC-1α immunopositivity evaluation.

**Fig 5:**
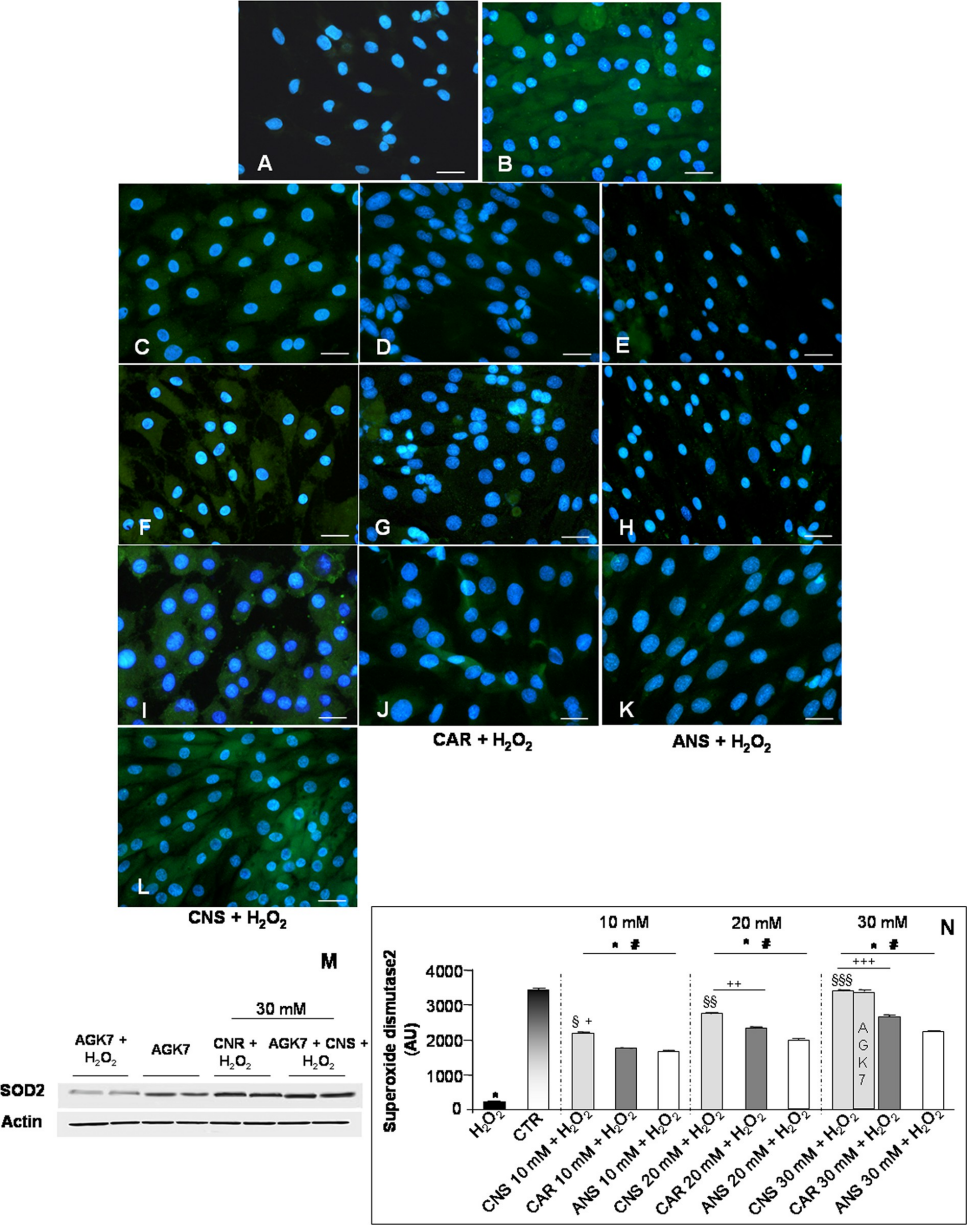
Superoxide dysmutase2 expression. Immunofluorescence photomicrographs of superoxide dysmutase2 (SOD2—green staining) of myotubes treated with hydrogen peroxide—H_2_O_2_ (A), untreated control myotubes—CTR (B), myotubes pre-incubated with carnosinol followed by H_2_O_2_-treatment—CNS + H_2_O_2_ (C, F, I), pre-incubated with carnosine followed by H_2_O_2_-treatment—CAR + H_2_O_2_ (D, G, J) and pre-incubated with anserine followed by H_2_O_2_-treatment—ANS + H_2_O_2_ (E, H, K) at the concentrations of 10 mM (C-E), 20 mM (F-H) and 30 mM (I-K) and myotubes incubated with AGK7 then with carnosinol (30 mM) and followed by H_2_O_2_-treatment—AGK7 + CNS + H_2_O_2_ (L). Bar equal: 20 μm. (M) Representative western blot showing SOD2 level in myotubes incubated with AGK7 and then with hydrogen peroxide—AGK7 + H_2_O_2_, myotubes incubated with only AGK7—AGK7, myotube pre-incubated with carnosinol (30 mM) followed by H_2_O_2_-treatment—CNS + H_2_O_2_ and myotubes incubated with AGK7 then with carnosinol (30 mM) and followed by H_2_O_2_-treatment—AGK7 + CNS + H_2_O_2_. (N) The graph summarizes the quantitative analysis of SOD2 immunopositivity. * p≤0.05 *vs* CTR; # p≤0.05 *vs* H_2_O_2;_ § p≤0.05 *vs* CAR + H_2_O_2_ 10 mM; §§ p≤0.05 *vs* CAR + H_2_O_2_ 20 mM; §§§ p≤0.05 *vs* CAR + H_2_O_2_ 30 mM; + p≤0.05 *vs* ANS + H_2_O_2_ 10 mM; ++ p≤0.05 *vs* ANS + H_2_O_2_ 20 mM and +++ p≤0.05 *vs* ANS + H_2_O_2_ 30 mM.

Regarding the antioxidant properties of the studied HCDs, we evaluated also the expression of the endogenous antioxidants SOD2 and CAT [21]. SOD2 was very weakly expressed in H_2_O_2_-treated cells (**Fig 5A**) as compared to untreated control cells that showed a moderate/strong expression (**Fig 5B**). The incubation of L6 cells with only carnosinol, carnosine or anserine at all the studied concentrations showed a moderate/strong expression of SOD2, comparable to that observed in the untreated control myotubes (**S1C Fig**). Remarkably, the pre-incubation of H_2_O_2_-treated cells with carnosinol showed a significant and concentration-dependent increased in the expression of this antioxidant enzyme (**Fig 5C, 5F and 5I**). In particular, the pre-incubation of H_2_O_2_-treated cells with carnosinol showed a higher SOD2 expression respect to concentration-matched H_2_O_2_-treated cells pre-incubated with carnosine (**Fig 5D, 5G and 5J**) or anserine (**Fig 5E, 5H and 5K**). Interestingly, myotubes incubated with AGK7 then with carnosinol (30 mM) and followed by H_2_O_2_-treatment maintained the moderate/strong SOD2 expression (**Fig 5L**), as observed in myotubes pre-incubated with carnosinol (30 mM) and then H_2_O_2_-treated. Furthermore, the myotube incubated with AGK7 and then treated with H_2_O_2_ showed no difference in SOD2 expression compared to the respective experimental group not treated with the SIRT3 inhibitor. However, myotubes incubated only with AGK7 showed a reduction of SOD2 expression (weak/moderate expression) respect to untreated cells. The above reported observations are confirmed also by western blot analyses (**Fig 5M**). **Fig 5N** summarized SOD2 immunopositivity evaluation.

**Fig 6:**
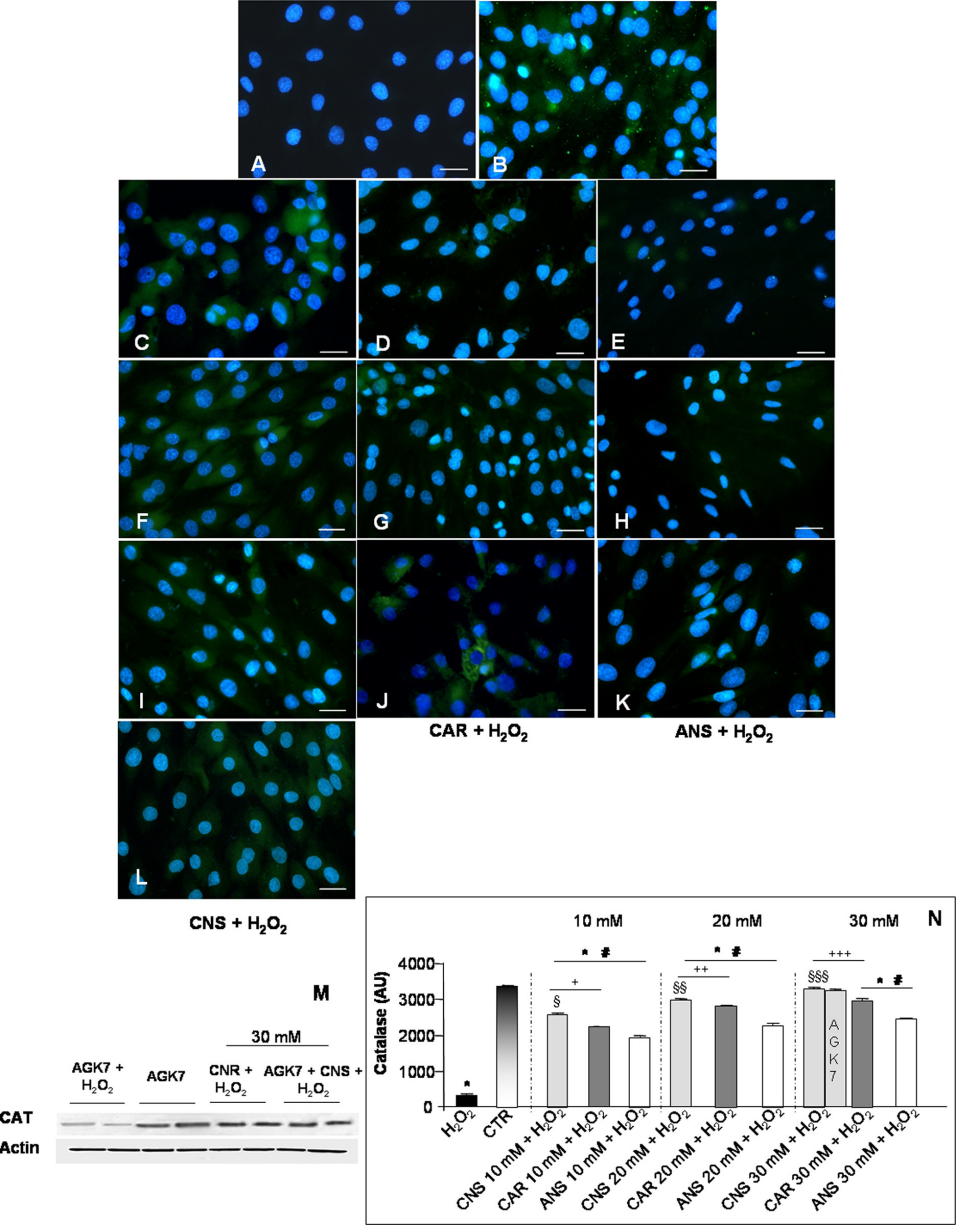
Catalase expression. Immunofluorescence photomicrographs of catalase (CAT—green staining) of myotubes treated with hydrogen peroxide—H_2_O_2_ (A), untreated control myotubes—CTR (B), myotubes pre-incubated with carnosinol followed by H_2_O_2_-treatment—CNS + H_2_O_2_ (C, F, I), pre-incubated with carnosine followed by H_2_O_2_-treatment—CAR + H_2_O_2_ (D, G, J) and pre-incubated with anserine followed by H_2_O_2_-treatment—ANS + H_2_O_2_ (E, H, K) at the concentrations of 10 mM (C-E), 20 mM (F-H) and 30 mM (I-K) and myotubes incubated with AGK7 then with carnosinol (30 mM) and followed by H_2_O_2_-treatment—AGK7 + CNS + H_2_O_2_ (L). Bar equal: 20 μm. (M) Representative western blot showing CAT level in myotubes incubated with AGK7 and then with hydrogen peroxide—AGK7 + H_2_O_2_, myotubes incubated only with AGK7—AGK7, myotube pre-incubated with carnosinol (30 mM) followed by H_2_O_2_-treatment—CNS + H_2_O_2_ and myotubes incubated with AGK7 then with carnosinol (30 mM) and followed by H_2_O_2_-treatment—AGK7 + CNS + H_2_O_2_. (N) The graph summarizes the quantitative analysis of CAT immunopositivity. * p≤0.05 *vs* CTR; # p≤0.05 *vs* H_2_O_2;_ § p≤0.05 *vs* CAR + H_2_O_2_ 10 mM; §§ p≤0.05 *vs* CAR + H_2_O_2_ 20 mM; §§§ p≤0.05 *vs* CAR + H_2_O_2_ 30 mM; + p≤0.05 *vs* ANS + H_2_O_2_ 10 mM; ++ p≤0.05 *vs* ANS + H_2_O_2_ 20 mM and +++ p≤0.05 *vs* ANS + H_2_O_2_ 30 mM.

The same pattern of expression was observed for CAT: H_2_O_2_-treated cells showed an absent/very weak CAT expression (**Fig 6A**) respect to a moderate/strong expression in untreated control myotubes (**Fig 6B**). As for SOD2 expression, L6 myotubes incubated only with each one of the three investigated HCDs showed the same moderate/strong CAT expression observed in untreated control cells (**S1D Fig**). Also CAT expression was significantly and in a concentration-dependent manner increased in H_2_O_2_-treated cells pre-incubated with carnosinol, carnosine or anserine (**Fig 6C–6K**) showing a higher expression in H_2_O_2_-treated cells pre-incubated with carnosinol (**Fig 6C, 6F and 6I**) respect to pre-incubation with carnosine (**Fig 6D, 6G and 6J**) or anserine (**Fig 6E, 6H and 6K**). As observed for SOD2, the AGK7 incubation of H_2_O_2_-treated myotubes also pre-incubated with carnosinol (30 mM) preserved the moderate expression of CAT (**Fig 6L**), which appeared comparable to H_2_O_2_-treated myotubes pre-incubated with carnosinol (30 mM) without SIRT3 inhibitor treatment. Furthermore, myotubes incubated only with AGK7 showed, as for SOD2, a decrease of CAT expression respect to untreated control cells. The above reported observations are confirmed also by western blot analyses (**Fig 6M)**. **Fig 6N** summarized CAT immunopositivity evaluation.

**Fig 7:**
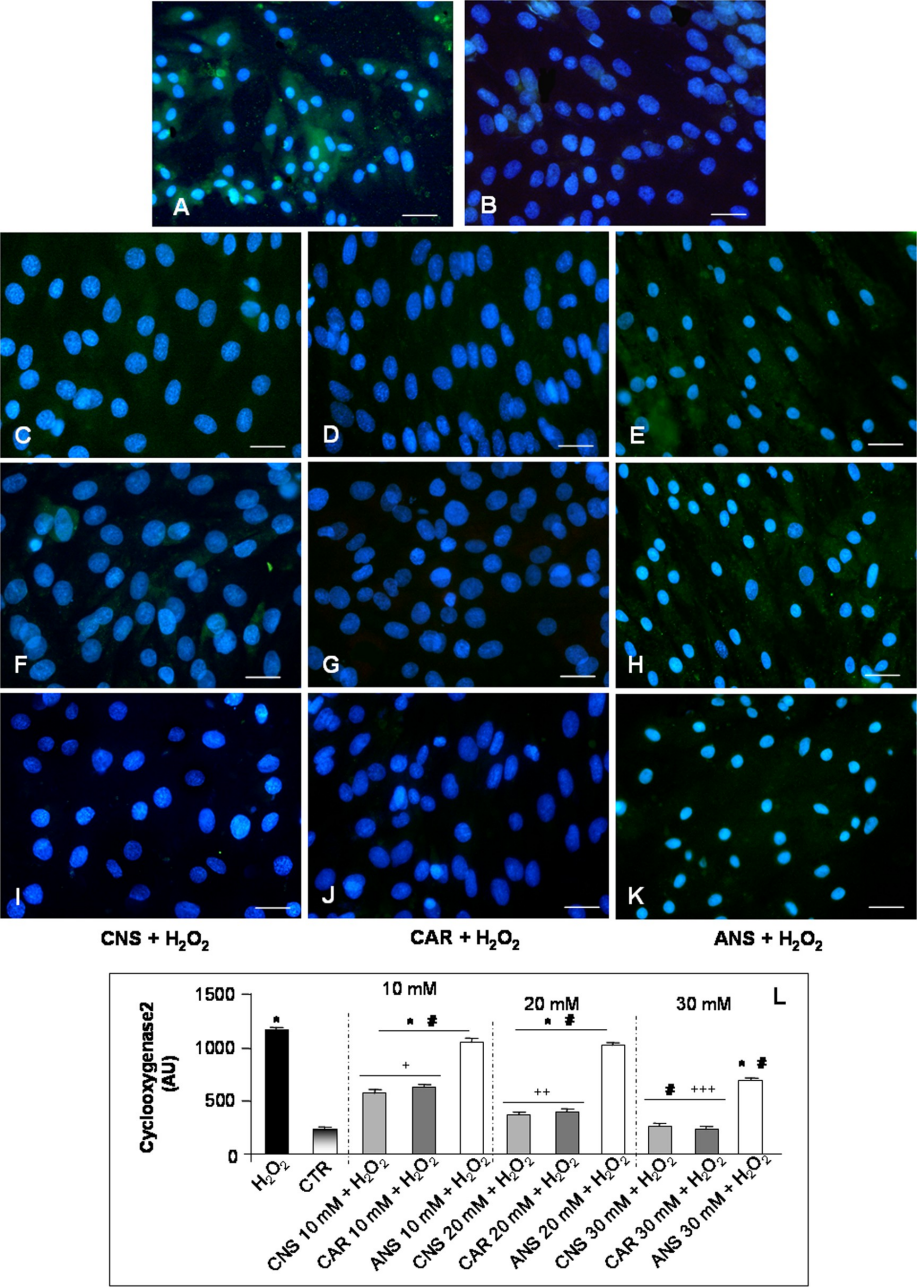
Cyclooxygenase2 expression. Immunofluorescence photomicrographs of cyclooxygenase2 (COX2—green staining) of myotubes treated with hydrogen peroxide—H_2_O_2_ (A), untreated control myotubes—CTR (B), myotubes pre-incubated with carnosinol followed by H_2_O_2_-treatment—CNS + H_2_O_2_ (C, F, I), pre-incubated with carnosine followed by H_2_O_2_-treatment—CAR + H_2_O_2_ (D, G, J) and pre-incubated with anserine followed by H_2_O_2_-treatment—ANS + H_2_O_2_ (E, H, K) at the concentrations of 10 mM (C-E), 20 mM (F-H) and 30 mM (I-K). Bar equal: 20 μm. (L) The graphs summarize the quantitative analysis of COX2 immunopositivity. * p≤0.05 *vs* CTR; # p≤0.05 *vs* H_2_O_2;_ § p≤0.05 *vs* CAR + H_2_O_2_ 10 mM; §§ p≤0.05 *vs* CAR + H_2_O_2_ 20 mM; §§§ p≤0.05 *vs* CAR + H_2_O_2_ 30 mM; + p≤0.05 *vs* ANS + H_2_O_2_ 10 mM; ++ p≤0.05 *vs* ANS + H_2_O_2_ 20 mM and +++ p≤0.05 *vs* ANS + H_2_O_2_ 30 mM.

H_2_O_2_-incubation induces the production of also inflammatory cytokines that could result, in turn, in muscle atrophy as previously reported from our and other groups [11, 22] and these changes are strongly associated with oxidative stress and apoptosis [23]. So, in the present study was investigated also the expression of the inducible inflammatory marker COX2 [24]. COX2 was moderately expressed in the H_2_O_2_-treated myotubes (**Fig 7A**), however, it was almost absent in the untreated control cells (**Fig 7B**). In L6 cells incubated only with carnosinol, carnosine or anserine at all the three investigated concentrations, COX2 expression was either absent or very weak (**S1E Fig**), as observed in the untreated control myotubes. Remarkably, pre-incubation of H_2_O_2_-treated cells with carnosinol (**Fig 7C, 7F and 7I**) or carnosine (**Fig 7D, 7G and 7J**) reduced significantly and in a concentration-dependent manner the expression of this inflammatory marker, resulting, at 30 mM, in an absent/very weak COX2 expression comparable to untreated control cells. However, anserine pre-incubation of H_2_O_2_-treated cells showed a moderate/weak COX2 expression (**Fig 7E, 7H and 7K**) higher than that observed in concentration-matched H_2_O_2_-treated cells pre-incubated with carnosinol or carnosine. **Fig 7L** summarized COX2 immunopositivity analyses.

## Discussion

In the present study, we investigated the role of carnosinol, a new carnosine analogue, against oxidative stress and inflammation induced by H_2_O_2_-treatment of L6 skeletal muscle cells *in vitro*. Its effects have been evaluated in comparison to carnosine and anserine and the findings are very interesting demonstrating that it is more efficacy respect to the other well-known HCDs.

Cell viability and morphology of L6 cells were greatly preserved in myotubes pre-incubated with carnosinol or carnosine and then exposed to oxidative stress respect to pre-incubation with anserine. Notably, the protective effects of carnosine were concentration-dependent and the carnosine rejuvenating effects in cultured human fibroblasts were more evident at the concentration of 50 mM [25]. In the present study, we observed beneficial antioxidant, anti-inflammatory and anti-apoptotic effects at lower HCDs concentrations (10–30 mM). Moreover, we observed a very strict link with the mitochondrial environment mediated by PGC-1α and SIRT3 expressions. A previous study has demonstrated that PGC-1α knockdown effectively reduces SIRT3 expression in muscle cells, hepatocytes and adipocytes [26]. Furthermore, Zhang et al. [20] observed a skeletal muscle restoration in chronic obstructive pulmonary disease due to activation and up-regulation of the PGC-1α/SIRT3 signaling pathway through curcumin supplementation, so PGC-1α and SIRT3 are important for the induction of radical oxygen species-detoxifying enzymes. Our findings are in agreement with these and other studies that showed an over-expression of SIRT3, which leads to SOD2 deacetylation and activation and it also down-regulates pro-inflammatory markers in L6 cells and L6-insulin-resistant cells [27, 28]. According to our data, PGC-1α and SIRT3 modulation represent novel targets to counteract oxidative stress-associated diseases. In fact, in the present study PGC-1α and SIRT3 expressions are significantly decreased in association with the increased skeletal muscle damage and oxidative stress response. Notably, SIRT3, localized in mitochondria, regulates energy homeostasis and oxidative metabolism in addition to oxidative stress and cellular injury [29]. Thereby, carnosine could decrease oxidative stress not only by direct scavenging of reactive species, but also by regulating mitochondrial function and, as recently observed, the antioxidative and mitochondria-protecting properties of carnosine play a primary role against lead-induced reproductive toxicity [30]. Furthermore, Nagasawa et al. [31], using both *in vitro* and *in vivo* experiments, demonstrated the effectiveness of carnosine to prevent both lipid and protein oxidation at skeletal muscle level. These previous observations and our study indicate that carnosine may act through its antioxidant activity playing an active role in cellular metabolism.

Regarding anserine, we showed that it has lower effects against H_2_O_2_-oxidative stress induction at skeletal muscle level *in vitro*. According to [32] anserine is a potent antioxidant, which activates the intracellular heat shock protein70/heme oxygenase-1 defence signalling against oxidative, and glycative stress on renal tubular cells *in vitro* and on kidney of diabetic mice. In particular, in human kidney, anserine levels are higher compared to carnosine [2] suggesting the important role of anserine in kidney. Our data are not contrasting with these findings because it is known that the rat skeletal muscles contain, as reported by [33] ca. 600 mg carnosine/100 wet weight and ca. 200 mg anserine/100 g, so the different distribution of HCDs in skeletal muscles and in kidney could reveal their organ-specific action. Moreover, we can hypothesize that anserine needs a higher concentration for having its activity.

The new finding of this study is the beneficial effects of carnosinol in comparison to carnosine and anserine. For evaluating the role of carnosinol, we incubated myotubes with a SIRT3 inhibitor. Interestingly, we observed that both SOD2 and CAT expressions are comparable to H_2_O_2_-treated myotube pre-incubated with only carnosinol (30 mM). This finding suggest that carnosinol’s effects are not blocked. However, Kong et al. [34] reported that induction of reactive oxygen species-detoxifying enzymes, like SODs, by PGC-1α was impaired by SIRT3 knockdown in myotubes. In the present study we showed that the endogenous detoxifying enzymes are preserved also after SIRT3 inhibition due to carnosinol, as carnosine, is a potent scavenger of reactive species and of byproducts of oxidative stress [7] and so preserves endogenous antioxidant defence. It is, however, important underline that SOD2 and CAT expressions are reduced in cells incubated only with SIRT3 inhibitor respect to untreated cells, confirming the link between PGC-1α and SIRT3. Moreover, carnosinol may also decrease/prevent oxidative stress through the modulation of PGC-1α mitochondrial signalling, which may act as a direct suppressor of reactive oxygen species production [35] and also as a downstream activator of SIRT3 in skeletal muscle [20]. Our data are in agreement with the latter findings due to PGC-1α is expressed also if SIRT3 is inhibited. Notably, Kong et al. [34] reported not only that PGC-1α induces SIRT3 expression, but also that SIRT3 stimulates PGC-1α in a strengthen feedback loop. These observations confirmed our data in which the incubation of myotubes with only SIRT3 inhibitor decrease PGC-1α expression, while, notably, its expression is preserved if myotubes are pre-incubated with carnosinol.

**Fig 8:**
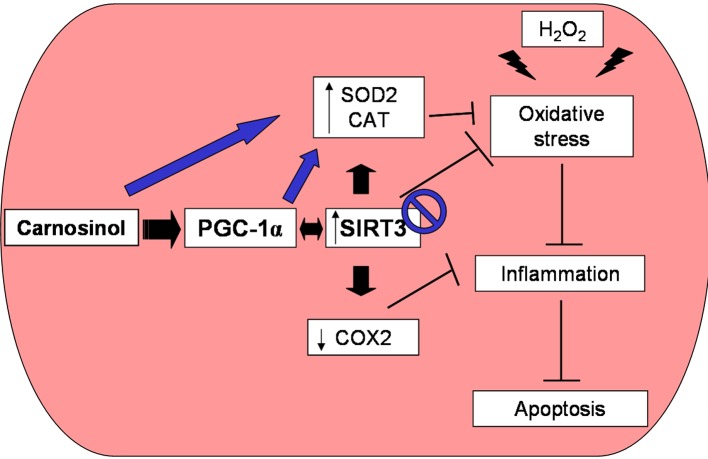
Carnosinol therapeutic potentials. Schematic representation of the therapeutic protective antioxidative effects of carnosinol against hydrogen peroxide-mediated alterations at myotube level showing that carnosinol may act through the mitochondrial PGC-1α/SIRT3 signaling pathway (black arrows), but if SIRT3 is inhibited carnosinol’s effects are not blocked (blue arrows). H_2_O_2_: hydrogen peroxide; CAT: catalase; COX2: cyclooxygenase2; PGC-1α: peroxisome proliferator-activated receptor gamma coactivator-1alpha; SIRT3: sirtuin3; SOD2: superoxide dysmutase2.

At our knowledge only very recently, Anderson et al. [7] demonstrated that carnosinol retained and surpassed the efficacy of carnosine on metabolic disorders of obesity and our current findings support and stress the effects of carnosinol indicating that they are due to its RCS-scavenging capacity and to its mitochondrial modulation. Importantly, the beneficial effects of carnosinol treatment on oxidative stress and inflammation were paralleled also to the improvements in metabolic parameters. As above reported, our data underlined that carnosinol is more efficient with respect to the other HCDs in the preservation and reduction of oxidative stress, apoptosis and inflammation (**Fig 8**).

We agree with the results of Anderson et al. [7] suggesting that carnosinol is the most promising carnosine derivate that has been synthesized at this point. In fact, it has high oral bioavailability and it is resistant to the hydrolytic action of carnosinases making it very stable respect to carnosine, so we can suggest that it represents a very promising molecules for metabolic diseases associated to oxidative stress and inflammation.

## Supporting information

S1 Fig The graphs showed the sirtuin3 (**A**), peroxisome proliferator-activated receptor gamma coactivator-1α (**B**), superoxide dysmutase2 (**C**), catalase (**D**) and cyclooxygenase2 (**E**) quantitative analyses, expressed in arbitrary units (AU), of myotubes treated with hydrogen peroxide—H_2_O_2_, control myotubes—CTR, myotubes pre-incubated with carnosinol—CNS, carnosine—CAR or anserine—ANS at the concentrations of 10 mM, 20 mM and 30 mM.
(TIF)
