# Supplementary figures and images for: A carnosine analog with therapeutic potentials in the treatment of disorders related to oxidative stress
Source: PLoS One. 2019 Apr 9;14(4):e0215170. doi: 10.1371/journal.pone.0215170 (PMC6456212; doi:10.1371/journal.pone.0215170)

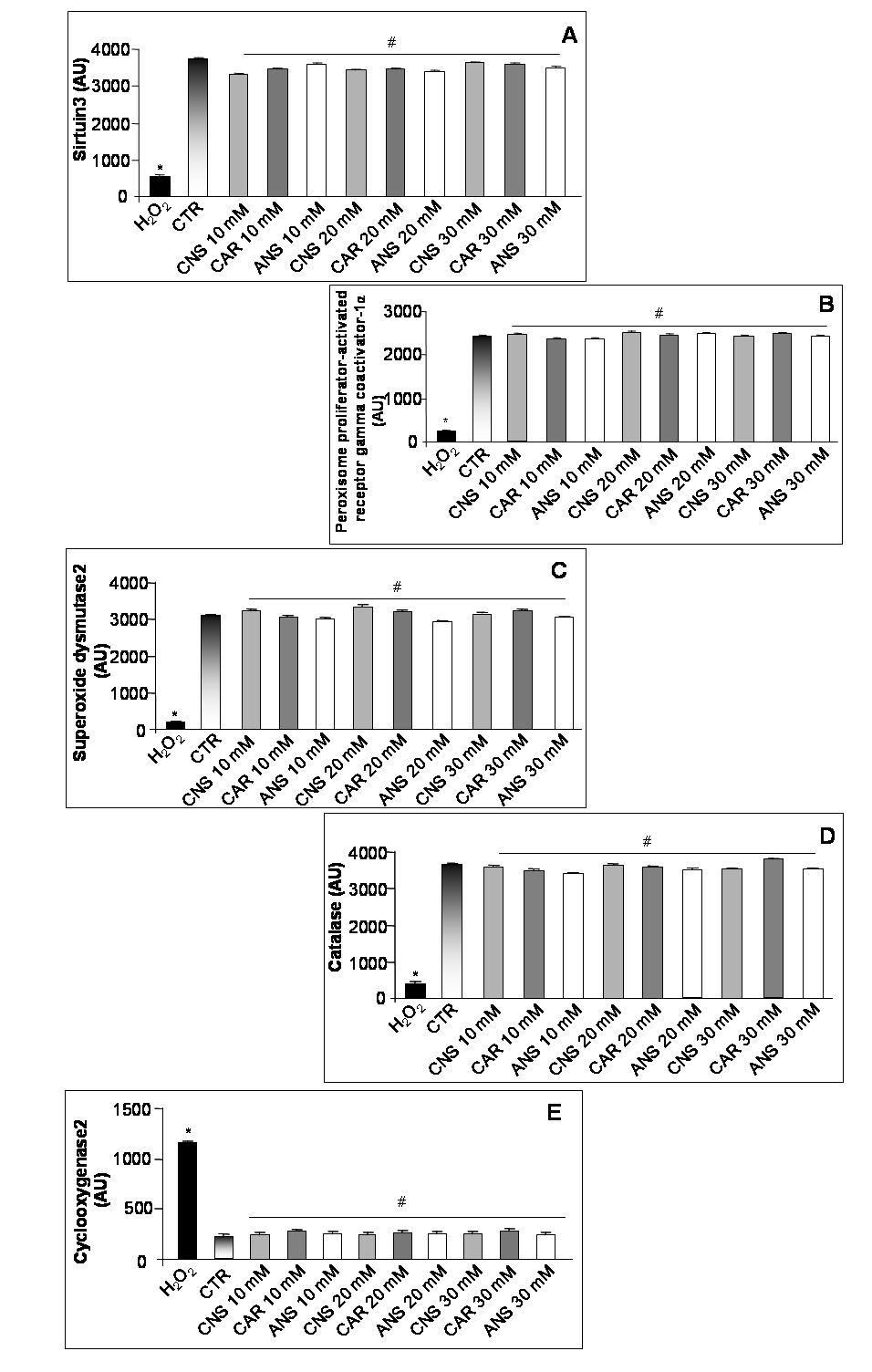

Supplement: S1 Fig — The graphs showed the sirtuin3 (A), peroxisome proliferator-activated receptor gamma coactivator-1α (B), superoxide dysmutase2 (C), catalase (D) and cyclooxygenase2 (E) quantitative analyses, expressed in arbitrary units (AU), of myotubes treated with hydrogen peroxide—H2O2, control myotubes—CTR, myotubes pre-incubated with carnosinol—CNS, carnosine—CAR or anserine—ANS at the concentrations of 10 mM, 20 mM and 30 mM. (TIF) [file pone.0215170.s001.tif]
